# Characteristics and Comparative Analysis of Six Mitogenomes of Genus *Kiefferulus* Goetghebuer, 1922 (Diptera: Chironomidae)

**DOI:** 10.3390/insects15090646

**Published:** 2024-08-28

**Authors:** Dan Zhang, Wei-Dong Jin, Hai-Feng Xu, Xue-Bo Li, Yong-Wei Jiang, Dai-Qing Li, Xiao-Long Lin

**Affiliations:** 1 Characteristic Laboratory of Forensic Science in Universities of Shandong Province, Shandong University of Political Science and Law, Jinan 250014, China; zhangdanioz2016@gmail.com (D.Z.); 000798@sdupsl.edu.cn (X.-B.L.); 2 National Engineering Laboratory for Lake Pollution Control and Ecological Restoration, Chinese Research Academy of Environmental Sciences, Beijing 100012, China; kindong.04@163.com; 3 Engineering Research Center of Environmental DNA and Ecological Water Health Assessment, Shanghai Ocean University, Shanghai 201306, China; haifengxu2024@gmail.com; 4 Shanghai Universities Key Laboratory of Marine Animal Taxonomy and Evolution, Shanghai Ocean University, Shanghai 201306, China; 5 College of Water Sciences, Beijing Normal University, Beijing 100875, China; jyw435@126.com; 6 Research Center for Eco-Environmental Sciences, Chinese Academy of Sciences, Beijing 100085, China

**Keywords:** Chironomidae, mitogenome, *Kiefferulus*

## Abstract

**Simple Summary:**

*Kiefferulus* Goetghebuer, 1922 is an important genus within Chironomidae, known for its roles in aquatic ecosystems. The studies of this genus have mainly focused on morphological aspects, and the molecular data, especially high-throughput sequencing, are seriously inadequate, limiting our understanding of its evolution history. Herein, we sequenced the mitogenomes of six *Kiefferulus* species and analyzed the characteristics of the mitochondrial genome among these species. Furthermore, we reconstructed the phylogenetic relationship of the genus *Kiefferulus* based on two different methods: Maximum likelihood and Bayesian inference. In addition, the phylogenetic analysis of *Kiefferulus* exhibited that six newly obtained species were obviously distinguished from others. Our study provides a critical foundation for future research on the evolutionary biology of Chironomidae.

**Abstract:**

Chironomidae is a cosmopolitan and species-rich family of insects, with many species serving as useful indicators of aquatic ecosystem health. In this study, we newly sequenced six species of *Kiefferulus* Goetghebuer, 1922 (Chironomidae: Chironominae) by high-throughput sequencing technology. We analyzed characters of the mitochondrial genome, including the sequence length, nucleotide composition, and evolutionary rates of this genus. The size of the newly obtained sequences ranged from 15,588 to 15,767 bp, and all of them included 22 tRNAs, 13 PCGs, 2 rRNAs, and 1 CR. The CR showed the highest AT content relative to the PCGs, rRNAs, and tRNAs. Relative synonymous codon usage analysis showed that UUA, UUU, and AUU are the preferred codons. The ratio of nonsynonymous (Ka) to synonymous (Ks) substitution rates showed that all Ka/Ks of PCGs were lower than 1, with *ATP8* having the highest evolution rate, while *COX1* exhibited the lowest evolution rate. We reconstructed the phylogenetic relationship of the genus *Kiefferulus* based on eight species (six ingroups and two outgroups), using five matrices and employing Maximum likelihood and Bayesian inference approaches. Phylogenetic analysis of the *Kiefferulus* showed that six species within this genus were classified into a monophyletic clade.

## 1. Introduction

Chironomidae, as a cosmopolitan and important aquatic insect family, have a high diversity with more than 7500 described species worldwide [1]. Some Chironomids exhibit considerable resilience and resistance to environmental stressors [1,2,3,4]. The larvae of Chironomidae inhabit a wide range of environments, including freshwater, terrestrial, and semi-aquatic habitats [4]. Many species are highly sensitive to changes in environmental conditions, such as trophic state temperature, salinity, or acidity, making them useful indicator organisms for aquatic ecosystems [5].

*Kiefferulus* Goetghebuer, 1922 is a genus within the tribe Chironomini of the family Chironomidae, and can be distinguished from other genera by its characteristic hypopygium of males [6]. The larvae of this genus live in sediments of small- to medium-sized waterbodies, demonstrating a high level of adaptability to various environmental conditions; species of this genus can be used to assess the environment quality of tropical aquatic ecosystems [7,8]. Despite their ecological significance, research on this group is still inadequate, particularly in terms of morphological descriptions and molecular phylogenetic studies.

Mitochondrial genomes serve as significant molecular markers and have typically been utilized in investigations about phylogeny, evolutionary history, speciation, and phylogeography within insect taxa [2,9,10]. The maternal mode of inheritance, along with a high mutation rate and the ease of obtaining mitochondrial genomes, makes them particularly advantageous for the above research [3,11]. The insect mitochondrial genome usually ranges from 14,000 to 20,000 bp in length [9], including 2 ribosomal RNAs (rRNA), 22 transfer RNAs (tRNAs), 13 protein-coding genes (PCGs), and 1 non-coding control region (CR) [12]. Furthermore, the structural characteristics of mitogenomes can offer additional insights and corroborating evidence for taxonomic classification [2,3], and the number of complete mitochondrial genomes of insects has significantly increased. This advancement, driven by the rapid development of next-generation sequencing technologies, aids in resolving structural comparisons and understanding the evolutionary history of different groups [12,13,14].

In recent times, an increasing number of studies on the mitochondrial genomes of Chironomidae have greatly advanced research in systematics and evolutionary history. Additionally, the growing availability of mitogenomes of Chironomidae offers valuable opportunities to investigate the structure and evolutionary patterns of their mitochondria [2,3,14,15]. For example, Lin and his associates utilized mitochondrial genomes to examine the taxonomic characteristics and phylogenetic relationships of Prodiamesinae within the Chironomidae [2]. Their findings revealed that Prodiamesinae is a group of Orthocladiinae, providing additional insights into the evolutionary history of this group [2]. However, resources for the mitogenomes of the genus *Kiefferulus* are limited. Currently, only one mitogenome is available for *Kiefferulus* [16], which restricts our understanding of the phylogenetic relationships and evolutionary history of this group.

In this study, we sequenced, assembled, and annotated six mitogenomes of species within the genus *Kiefferulus* and analyzed their mitogenomic characters including their main features, evolutionary rates, and substitutions. In addition, we used eight mitochondrial genomes to reconstruct the phylogenetic relationships of *Kiefferulus* species. The results not only expand our understanding of the mitogenomic features of Chironomidae and *Kiefferulus* but also provide new information for the definition of the genus *Kiefferulus.*

## 2. Materials and Methods

### 2.1. Taxon Sampling and Sequencing

In this study, six species of the genus *Kiefferulus* were selected for sequencing analysis (Table 1). Cranston et al. (1990) synonymized the genera *Nilodorumi* Kieffer and *Carteronica* Kieffer with the genus *Kiefferulus* [17].Fang et al. reported the first mitogenome of the genus *Kiefferulus*; however, they mistakenly used the species name *Nilodorum tainanus* [16]. Thus, we used our newly sequenced data of *K. tainanus* for our analyses. In addition, we selected two species of *Chironomus (Chironomus kiiensis*: NC_069311.1; *Chironomus plumosus*: NC_069032.1) [16,18], which were closely related to the genus *Kiefferulus* as outgroups, based on a prior study of Chironomidae [6].

Herein, newly obtained samples were collected from China, Namibia, and New Caledonia during 2013–2021 (see detailed information in Table 1). All samples were deposited in 95% ethanol at −20 °C firstly for morphological examination and DNA extraction. X.L.L. identified all specimens, and all vouchers were stored at the College of Fisheries and Life, Shanghai Ocean University, Shanghai, China.

Qiagen DNeasy Blood & Tissue Kit was used to extract the whole-genomic DNA based on the manufacturer’s protocol. Qubit^®^ 2.0 Flurometer (ThermoFisher, Waltham, MA, USA) was used to measure the DNA concentration with Qubit^®^ DNA Assay Kit, and all the whole-genome DNA was then sent to the sequencing company (Berry Genomics, Beijing, China).

Sequencing libraries were generated by the Truseq Nano DNA HT sample preparation Kit (Illumina, San Diego, CA, USA), and the Illumina NovaSeq 6000 platform was used to sequence all raw data, generating 150 bp paired-end reads with an insert size of 350 bp. Short reads, low-quality reads, and adapters were removed from the raw data by Trimmomatic v0.32 (Jülich, Germany) [19].

### 2.2. Assembly, Annotation, and Composition Analyses

To guarantee precision, we employed two techniques for de novo assembly: (1) NOVOPlasty v3.8.3 (Brussel, Belgium) [20] was used to assemble the mitogenome with a k-mer size of 23–39 bp and (2) IDBA-UD v1.1.3 (Boston, MA, USA) [21] was performed to assemble with a “–mink 40 –maxk 120” parameter. Geneious 2020.2.1 [22] was employed to analyze and compare the mitogenome sequences obtained through the aforementioned methods and subsequently merge them into a single sequence. Annotation and analysis for the secondary structure of tRNAs were performed by tRNAscan SE 2.0 [23] and MITOS WebServer. MEGA X [24] was used to analyze the base composition and usage of mitogenomes. SeqKit v0.16.0 (Chongqing, China) [25] was utilized to evaluate the bias of the nucleotide composition of each gene. The AT-skew and GC-skew were computed as follows: AT-skew = (A − T)/(A + T), and GC-skew = (G − C)/(G + C). MEGA X was performed to calculate the AT-skew, GC-skew, and the relative synonymous codon usage (RSCU) of the newly sequenced species.

The rates of nonsynonymous substitution (Ka) and synonymous substitution (Ks) for each PCG were calculated using DnaSP 6.0 [26]. The online server CGview (https://cgview.ca/, accessed on 22 June 2024) was utilized to create the mitogenome map, which illustrates sequence features.

### 2.3. Phylogenetic Relationship

To reconstruct the phylogenetic relationships of the genus *Kiefferulus*, we used 2 rRNA and 13 PCG genes of *Kiefferulus* mitogenomes. MAFFT v7.450 (Osaka, Japan) [27] was applied to align the nucleotide and protein sequences with the L-INS-I method. Trimal v1.4.1 (Barcelona, Spain) [28] was used to trim sequences under the “-automated1” strategy. Finally, we generated five matrices to reconstruct the phylogenetic relationship of *Kiefferulus*, and FASconCAT-G v1.04 (Santa Cruz, CA, USA) [29] was applied to concatenate matrices: the (1) cds_rrna matrix included all PCGs and two rRNA nucleotide reads; (2) cds_faa matrix contained all PCG amino acid reads; (3) cds12_fna matrix contained all PCG nucleotide reads except the third codon positions; (4) cds_fna matrix included all PCG nucleotide reads; and (5) cds12_rrna matrix contained PCG nucleotide reads which removed the third codon positions and two rRNA genes.

We used Bayesian inference (BI) and Maximum likelihood (ML) to infer the phylogenetic relationships of the genus *Kiefferulus* for all matrices. In all ML analyses, the best-fitting substitution models were selected via ModelFinder [30] within IQ-TREE 2 (Canberra, ACT, Australia) [31]. In addition, to minimize the impact of long-branch attraction, we used the posterior mean site frequency (PMSF) [32] model with the ‘-m − mtART + C60 + FO + R’ command to reconstruct the phylogenetic relationship of the genus *Kiefferulus* based on the matrix cds_faa. BI trees were implemented via PhyloBayes-MPI (Montréal, QC, Canada) [33] with the site-heterogeneous mixture model ‘-m CAT + GTR’. For the BI analysis, the Markov chain Monte Carlo (MCMC) analysis was performed twice with 10,000,000 generations, while maxdiff < 0.3 stopped the MCMC analysis. A consensus tree was created by combining the remaining trees after discarding 25% of the initial trees from each run as burn-in. Finally, we used iTOL, an online website, to beautify all trees (https://itol.embl.de/upload.cgi, accessed on 15 May 2024).

## 3. Results and Discussions

### 3.1. Mitogenomic Structure

**Figure 1:**
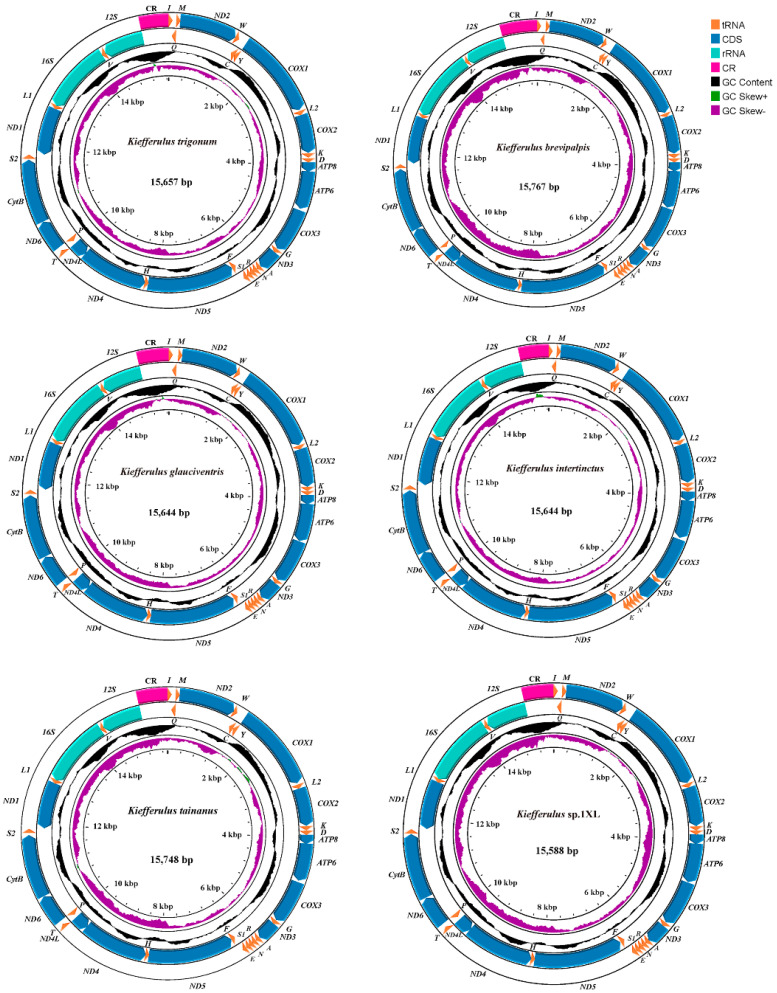
A mitogenome map showing the representative species from six species within the genus Kiefferulus. The arrow indicates the direction of gene transcription. We used normative abbreviations to represent PCGs and rRNAs, and single-letter abbreviations were used to represent tRNAs. The GC content of the complete mitogenome is shown in the second circle. The GC-skew of the complete mitogenome is shown in the third circle. The innermost circle shows the length of the complete mitogenome.

The Illumina NovaSeq 6000 platform generated 3 Gb raw reads for each sample. A total of six newly sequenced mitogenomes of the genus *Kiefferulus* were obtained here, and all of them were complete. All newly sequenced mitochondrial genomes were submitted in GenBank as PP884095–PP884099 and PP972215 (Table 1). The complete mitogenome of newly obtained species ranged from 15,588 (*Kiefferulus* sp.1XL) to 15,767 (*Kiefferulus brevipalpis*) bp in length; the unstable size of the CR was the main reason for the variation in mitogenome length (Table 2). As with all published insect mitochondrial genomes, all newly obtained sequences contained 37 typical genes, including 13 PCGs, 22 tRNAs, and 2 rRNAs (Figure 1). In general, the mitogenomic structure and nucleotide composition of newly obtained sequences displayed the typical characteristics of the Chironomidae [15,34,35]. The mitogenome characters of the newly sequenced species are shown in Figure 1.

The nucleotide composition of the newly obtained species exhibited similarities (Table 2), demonstrating the characteristic AT-biased composition that is commonly observed in Chironomidae and other insects [15,17,36]. The AT content of the newly obtained sequences ranged from 75.12% (*Kiefferulus* sp.1XL) to 79.44% (*Kiefferulus trigonum*), and the GC content ranged from 20.56% (*K. trigonum*) to 24.88% (*K*. sp.1XL). The AT-skew of all newly obtained mitogenomes was positive, while GC-skew was negative. The AT-skew of the newly sequenced mitogenomes ranged from 0.019 (*K. trigonum*) to 0.040 (*K. brevipalpis*), and the GC-skew ranged from −18.62 (*Kiefferulus tainanus*) to −0.186 (*K. trigonum*) (detailed information shown in Table 2).

### 3.2. Protein-Coding Genes, Codon Usage, and Evolutionary Rates

No significant disparities were observed in the size of tRNA, PCGs, and rRNAs among each species. The length of the 13 PCGs ranged from 11,220 (*K. tainanus*) to 11,621 (*K. trigonum*) bp. The AT content ranged from 73.10% (*K.* sp.1XL) to 78.24% (*K. trigonum*), while the GC content ranged from 21.76% (*K. trigonum*) to 26.90% (*K.* sp.1XL). By comparing and integrating previous research on Chironomidae, we found that the AT content at the third site of PCGs was notably higher than at the first and second sites [15] (Table 2). The skew metrics of all PCGs showed that both the AT-skew and GC-skew were negative. The AT-skew ranged from −0.192 (*K. tainanus*) to −0.175 (*K. brevipalpis*), and *K. tainanus* exhibited the highest GC-skew (1.38), while *Kiefferulus* sp.1XL showed the lowest GC-skew (−0.084) (for detailed information, see Table 2).

The CR size of the newly reported species ranged from 518 (*K. trigonum*) to 674 bp (*K. brevipalpis*). All 13 PCGs of the newly assembled mitogenomes exhibited the start codon ATN, which was typical for insect mitogenomes [9]. However, we identified several different start codon patterns: the start codon of *ATP6*, *COX2*, *COX3*, *CYTB*, *ND4*, and *ND4L* in five species was ATG; *ATP8* was ATT in two species;; *ND2* and *ND6* were ATT in five species; *COX1* and *ND1* were TTG in five species; *ND5* was GTG in one species; and so on, and for detailed information, see Figure S1. Although TAG or TAA were the common stop codons for this group, there were still several special stop codon patterns: ND4 had T as the stop codon in four species, and *ND3* had TAG as the stop codon in one species. More detailed information is shown in Figure S2.

**Figure 2:**
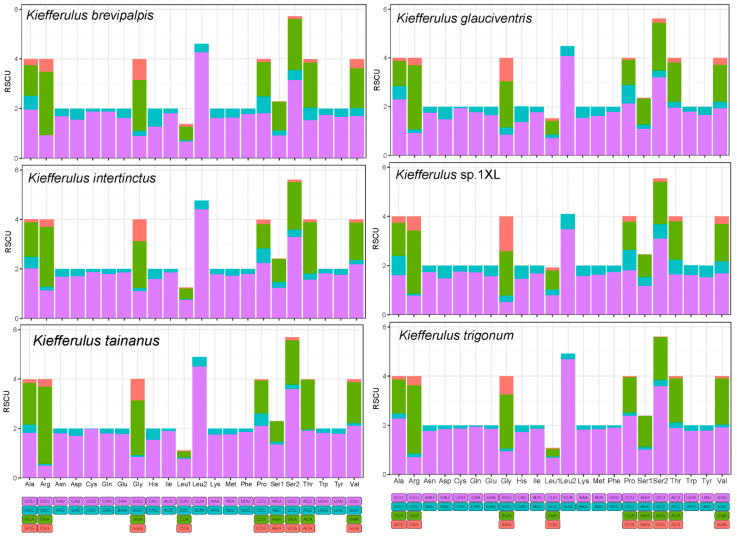
The relative synonymous codon usage (RSCU) of the mitochondrial protein-coding genes of five *Kiefferulus* species. The X-axis shows different amino acids, and the Y-axis shows the RSCU value (the number of times a certain synonymous codon is used/the average number of times that all codons coding the amino acid are used).

The relative synonymous codon usage (RSCU) patterns for the six mitochondrial genomes were predominantly congruent, as illustrated in Figure 2. The figure shows the RSCU values for all synonymous codons corresponding to the 22 amino acids, utilizing the 62 available codons in the 13 PCGs of *Kiefferulus* species. For the newly obtained *Kiefferulus* species, we found that the UUA, UUU, and AUU were the preferred codons. At the same time, Leu2, IIe, and Phe are frequently utilized amino acids, exhibiting the nucleotide composition preference for A/T. The codons RSCU > 2 of all newly sequenced species were as follows: UUA > UCU > CGA (Figure 2, Tables S1–S6).

**Figure 3:**
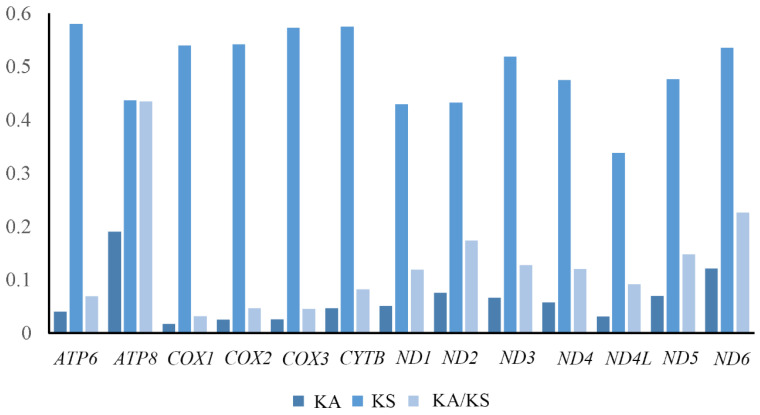
The evolution rate of 13 PCGs of *Kiefferulus*. Ka refers to nonsynonymous nucleotide substitutions, Ks refers to synonymous nucleotide substitutions, and Ka/Ks refers to the selection pressure of each PCG. The abscissa represents the 13 PCGs, and the ordinate represents Ka/Ks values.

The ratio of nonsynonymous (Ka) to synonymous (Ks) substitutions can be employed to gauge the proportion of non-neutral alterations compared to neutral ones, offering insights into the selective pressures acting on a protein-coding gene (PCG) [37,38]. In this study, the Ka/Ks of all PCGs was significantly lower than 1, ranging from 0.031 to 0.435. The evolution rates of the 13 PCGs were as follows: *ATP8* > *ND6* > *ND2* > *ND5* > *ND3* > *ND4* > *ND1* > *ND4L* > *CYTB* > *ATP6* > *COX2* > *COX3* > *COX1* (Figure 3). Each PCG exhibited different levels of purifying selection. *ATP8*, *ND6*, and *ND2* showed higher ω values, indicating relatively relaxed purifying selection pressure. Meanwhile, *COX1* and *COX3* exhibited hardly purifying selection.

### 3.3. Phylogenetic Relationships

**Figure 4:**
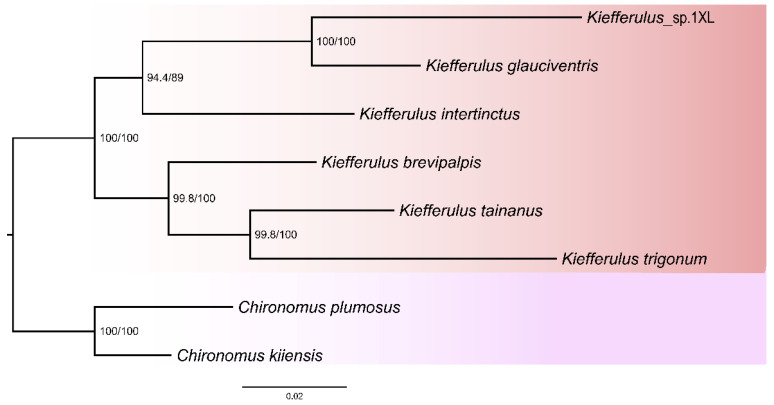
Phylogenetic tree of genus *Kiefferulus* based on cds_faa matrix with partition model in IQ-TREE. Support values on nodes indicate SH-aLRT/UFBoot2.

We used five matrices cds_faa (3726 sites), cds_fna (11,178 sites), cds_rrna (13,359 sites), cds12_fna (7452 sites), and cds12_rrna (9633 sites) to infer the phylogenetic relationship of the genus *Kiefferulus* based on ML and BI approaches. Two different methods based on five matrices yielded 11 trees (Figure 4 and Figures S3–S12). All trees exhibited highly consistent topology. In our phylogenetic results, six *Kiefferulus* were distinctively differentiated from others and grouped in a monophyletic clade. The phylogenetic results indicated that *K. trigonum* was close to *K. tainanus*, and *K. glauciventris* was close to *K.* sp.1XL (Figure 4 and Figures S3–S12).

The phylogenetic relationships of *Kiefferulus* and its close group are still controversial. Sæther [39] analyzed the phylogenetic relationships of the tribe Chironomini using the characters of females and found that the *Chironomus* is the sister group of *Kiefferulus*. Martin [40] reconstructed the phylogenetic relationship among genera closely related to *Chironomus* based on molecular data; the results well supported the broader *Kiefferulus* concept, with *Einfeldia* identified as the sister group of the genus *Kiefferulus*. Song et al. [6] used two ribosomal genes and four protein-coding gene segments to reconstruct the phylogenetic relationship of *Kiefferulus*. In addition, Tang et al. [41] also analyzed the relationship among *Kiefferulus* and its closely related genera, but their relationships have not been fully resolved. Unfortunately, due to limited sample availability, we have not yet resolved the phylogenetic relationships among *Kiefferulus* and its closely related groups. In future studies, researchers can include more sampling of genera, both adults and larvae, using more efficient data to explore the evolutionary history of this group, such as the low-coverage whole-genome genome, which shows great promise for resolving relations in difficult groups [42,43].

## 4. Conclusions

This study successfully obtained the complete mitogenomes of six *Kiefferulus* species: *K*. sp.1XL, *K. brevipalpis*, *K. glauciventris*, *K. intertinctus*, *K. tainanus*, and *K. trigonum*. The structural characteristics of all newly obtained sequences were similar to published Chironomidae species. The base composition calculated results showed that A and T were obviously biased for all newly obtained mitogenomes, which were also similar to other published Chironomidae species. The nonsynonymous-to-synonymous substitution ratio showed that *ATP8* displayed the highest evolution rate, and *COX1* had the lowest evolution rate. Thus, our study provided more available mitogenome data for the study of Chironomini and Chironomidae. Our phylogenetic results among the species of the genus *Kiefferulus* showed that all trees exhibited highly consistent topology, and six *Kiefferulus* were distinctively differentiated from others and grouped in a monophyletic clade.

## Figures and Tables

**Table 1:** Collection information of newly sequenced species in this study.

Species	Location	Longitude and Latitude	Elevation (m)	Life Stage	Date	Collector	Accession Number	References
*Kiefferulus* sp.1XL	Namibia, Otjozondjupa	16.745° E, −22.111° N	1220	Adult	4 December 2018	Xiaolong Lin	PP884096	This study
*Kiefferulus brevipalpis*	Namibia, Otjozondjupa	16.745° E, −22.111° N	1220	Adult	4 December 2018	Xiaolong Lin	PP884099	This study
*Kiefferulus glauciventris*	China, Xishuangbanna	99.989° E, 22.055° N	700	Adult	6 May 2013	Xiaolong Lin	PP884098	This study
*Kiefferulus intertinctus*	New Caledonia, Tontouta	166.206° E, −22.008° N	10	Pupa	11 May 2018	Nathalie Mary	PP884097	This study
*Kiefferulus tainanus*	China, Guangzhou	113.959° E, 23.339° N	149	Adult	12 April 2018	Xiaolong Lin	PP972215	This study
*Kiefferulus trigonum*	China, Huanggang	115.738° E, 31.095° N	552	Adult	11 July 2021	Shuang Qiu	PP884095	This study

**Table 2:** Nucleotide composition of newly sequenced species.

Species	Regions	Length (bp)	A	T	C	G	AT	GC	AT-Skew	GC-Skew
*Kiefferulus brevipalpis*	Whole genome	15,767	40.21	37.12	13.64	9.02	77.33	22.66	0.040	−0.204
	PCGs	11,619	31.22	44.50	12.85	11.43	75.72	24.27	−0.175	−0.059
	Site 1	4141	31.93	38.64	12.07	17.35	70.57	29.43	−0.090	0.155
	Site 2	3739	21.47	48.55	18.01	11.95	70.02	29.96	−0.385	−0.203
	Site 3	3739	40.26	46.30	8.46	4.98	86.56	13.44	−0.069	−0.233
	tRNA	1488	41.06	38.64	8.67	11.63	79.70	20.30	0.030	0.146
	l-rRNA	1381	41.20	43.95	4.71	10.14	85.16	14.84	−0.032	0.366
	s-rRNA	815	42.82	40.61	5.40	11.17	83.44	16.56	0.026	0.348
	CR	674	46.44	49.11	2.67	1.78	95.55	4.45	−0.028	−0.200
*Kiefferulus glauciventris*	Whole genome	15,644	39.24	37.77	13.84	9.15	77.01	22.99	0.019	−0.204
	PCGs	11,619	30.48	44.62	13.10	11.80	75.10	24.90	−0.188	−0.052
	Site 1	4141	31.41	37.34	13.13	18.12	68.75	31.25	−0.080	0.161
	Site 2	3739	21.45	48.57	18.04	11.93	70.02	29.98	−0.385	−0.206
	Site 3	3739	38.59	47.94	8.12	5.34	86.53	13.47	−0.108	−0.200
	tRNA	1487	41.29	38.53	8.88	11.30	79.83	20.17	0.035	0.120
	l-rRNA	1368	43.64	41.08	4.68	10.60	84.72	15.28	0.030	0.388
	s-rRNA	810	42.10	41.23	5.43	11.23	83.33	16.67	0.010	0.348
	CR	568	49.47	45.77	2.64	2.11	95.25	4.75	0.039	−0.111
*Kiefferulus intertinctus*	Whole genome	15,644	39.87	38.24	13.05	8.84	78.11	21.89	0.021	−0.192
	PCGs	11,619	31.13	45.34	12.33	11.20	76.47	23.53	−0.186	−0.048
	Site 1	4141	31.76	38.38	12.10	17.75	70.14	29.86	−0.088	0.185
	Site 2	3739	21.48	48.09	18.44	11.98	69.58	30.42	−0.379	−0.213
	Site 3	3739	40.16	49.54	6.44	3.86	89.70	10.30	−0.104	−0.235
*Kiefferulus intertinctus*	tRNA	1490	41.07	39.13	8.46	11.34	80.20	19.80	0.024	0.146
	l-rRNA	1376	42.08	43.31	4.65	9.96	85.39	14.61	−0.014	0.363
	s-rRNA	815	42.58	41.23	5.40	10.80	83.80	16.20	0.016	0.333
	CR	533	43.71	51.78	1.88	2.63	95.50	4.50	−0.085	0.167
*Kiefferulus* sp.1XL	Whole genome	15,588	38.42	36.70	15.03	9.85	75.12	24.88	0.023	−0.208
	PCGs	11,619	29.94	43.16	14.58	12.32	73.10	26.90	−0.181	−0.084
	Site 1	4141	31.84	36.34	13.80	18.02	68.18	31.82	−0.062	0.115
	Site 2	3739	21.19	48.13	18.61	12.06	69.33	30.67	−0.385	−0.214
	Site 3	3739	36.79	45.00	11.32	6.89	81.79	18.21	−0.100	−0.256
	tRNA	1488	40.39	38.98	9.07	11.56	79.37	20.63	0.018	0.121
	l-rRNA	1369	43.83	40.32	5.26	10.59	84.15	15.85	0.042	0.336
	s-rRNA	811	40.57	41.43	5.67	12.33	82.00	18.00	−0.010	0.370
	CR	546	48.17	46.34	3.48	2.01	94.51	5.49	0.019	−0.267
*Kiefferulus trigonum*	Whole genome	15,657	40.47	38.97	12.19	8.37	79.44	20.56	0.019	−0.186
	PCGs	11,621	31.82	46.42	11.21	10.54	78.24	21.76	−0.187	−0.031
	Site 1	4141	32.52	39.19	11.40	16.88	71.72	28.28	−0.090	0.163
	Site 2	3740	21.28	49.01	17.84	11.87	70.29	29.71	−0.392	−0.205
	Site 3	3740	41.67	51.05	4.40	2.87	92.72	7.28	−0.101	−0.180
	tRNA	1484	41.98	38.88	8.09	11.05	80.86	19.14	0.038	0.155
	l-rRNA	1378	41.36	43.90	4.57	10.16	85.27	14.73	−0.030	0.379
	s-rRNA	812	44.09	40.76	5.30	9.85	84.85	15.15	0.039	0.301
	CR	518	44.98	51.16	2.70	1.16	96.14	3.86	−0.064	−0.400
*Kiefferulus tainanus*	Whole genome	15,748	40.15	38.67	12.56	8.62	78.82	21.18	0.018	−18.620
	PCGs	11,220	30.77	45.41	11.75	12.08	76.18	23.82	−0.192	1.380
	Site 1	3740	31.34	38.05	11.9	18.72	69.39	30.61	−0.096	22.27
	Site 2	3740	20.43	47.51	18.96	13.10	67.94	32.06	−0.398	−18.27
	Site 3	3740	40.53	50.67	4.39	4.41	91.20	8.80	−0.111	0.300
	tRNA	1494	41.5	38.76	8.43	11.31	80.25	19.75	0.034	14.580
	l-rRNA	1388	43.23	42.51	4.54	9.73	85.73	14.27	0.008	36.360
	s-rRNA	814	43.24	41.28	5.16	10.32	84.52	15.48	0.023	33.330
	CR	554	45.67	49.28	3.43	1.62	94.95	5.05	−0.038	−35.710

## Data Availability

The following information was supplied regarding the availability of DNA sequences: the new mitogenomes are deposited in GenBank of NCBI, and the accession numbers are PP884095–PP884099 and PP972215.

## Supplementary Materials

The following supporting information can be downloaded at https://www.mdpi.com/article/10.3390/insects15090646/s1, Table S1. Relative synonymous codon usages (RSCUs) of PCGs of *Kiefferulus brevipalpis.* Table S2. Relative synonymous codon usages (RSCUs) of PCGs of *Kiefferulus glauciventris*. Table S3. Relative synonymous codon usages (RSCUs) of PCGs of *Kiefferulus intertinctus*. Table S4. Relative synonymous codon usages (RSCUs) of PCGs of *Kiefferulus* sp.1XL. Table S5. Relative synonymous codon usages (RSCUs) of PCGs of *Kiefferulus trigonum*. Table S6. Relative synonymous codon usages (RSCUs) of PCGs of *Kiefferulus tainanus*. Figure S1. Start codons of protein-coding genes among *Kiefferulus* mitogenomes. Figure S2. Stop codons of protein-coding genes of *Kiefferulus* mitogenomes. Figure S3. Bayesian inference phylogenetic tree of *Kiefferulus* based on analysis cd_faa with GTR + CAT model in phylobayes. Support values on nodes indicate Bayesian posterior probabilities. Figure S4. Maximum likelihood phylogenetic tree of *Kiefferulus* based on analysis cds_faa with PMSF model in IQTREE. Support values on nodes indicate SH-aLRT/UFBoot2. Figure S5. Bayesian inference phylogenetic tree of *Kiefferulus* based on analysis cd_fna with GTR + CAT model in phylobayes. Support values on nodes indicate Bayesian posterior probabilities. Figure S6. Maximum likelihood phylogenetic tree of *Kiefferulus* based on analysis cds_fna with a partitioned model in IQTREE. Support values on nodes indicate SH-aLRT/UFBoot2. Figure S7. Bayesian inference phylogenetic tree of *Kiefferulus* based on analysis cd_rrna with GTR + CAT model in phylobayes. Support values on nodes indicate Bayesian posterior probabilities. Figure S8. Maximum likelihood phylogenetic tree of *Kiefferulus* based on analysis cds_rrna with partitioned model in IQTREE. Support values on nodes indicate SH-aLRT/UFBoot2. Figure S9. Bayesian inference phylogenetic tree of *Kiefferulus* based on analysis cd12_fna with GTR + CAT model in phylobayes. Support values on nodes indicate Bayesian posterior probabilities. Figure S10. Maximum likelihood phylogenetic tree of *Kiefferulus* based on analysis cds12_fna with partitioned model in IQTREE. Support values on nodes indicate SH-aLRT/UFBoot2. Figure S11. Bayesian inference phylogenetic tree of *Kiefferulus* based on analysis cd12_rrna with GTR + CAT model in phylobayes. Support values on nodes indicate Bayesian posterior probabilities. Figure S12. Maximum likelihood phylogenetic tree of *Kiefferulus* based on analysis cds12_rrna with partitioned model in IQTREE. Support values on nodes indicate SH-aLRT/UFBoot2.
