# Supplementary material for: Characteristics and Comparative Analysis of Six Mitogenomes of Genus Kiefferulus Goetghebuer, 1922 (Diptera: Chironomidae)
Source: Insects. 2024 Aug 28;15(9):646. doi: 10.3390/insects15090646 (PMC11432734; doi:10.3390/insects15090646)
Supplement: Supplementary file 1 [file insects-15-00646-s001.zip › insects-3131479-supplementary.pdf]

Table S1 Relative synonymous codon usages (RSCUs) of PCGs of *Kiefferulus brevipalpis*

Codon	Count	RSCU	Codon	Count	RSCU
UUU	393	1.78	AUU	332	1.82
UUC	49	0.22	AUC	32	0.18
UUA	408	4.27	AUA	177	1.65
UUG	32	0.34	AUG	37	0.35
UCU	127	3.16	ACU	69	1.54
UCC	16	0.4	ACC	23	0.51
UCA	83	2.06	ACA	81	1.81
UCG	4	0.1	ACG	6	0.13
UAU	117	1.68	AAU	186	1.7
UAC	22	0.32	AAC	33	0.3
UAA*	0	0	AAA	83	1.63
UAG*	0	0	AAG	19	0.37
UGU	33	1.89	AGU	37	0.92
UGC	2	0.11	AGC	8	0.2
UGA	84	1.75	AGA	47	1.17
UGG	12	0.25	AGG	0	0
CUU	65	0.68	GUU	80	1.71
CUC	6	0.06	GUC	15	0.32
CUA	52	0.54	GUA	75	1.6
CUG	10	0.1	GUG	17	0.36
CCU	59	1.82	GCU	81	1.96
CCC	22	0.68	GCC	23	0.56
CCA	45	1.38	GCA	51	1.24
CCG	4	0.12	GCG	10	0.24
CAU	48	1.28	GAU	53	1.56
CAC	27	0.72	GAC	15	0.44
CAA	69	1.89	GAA	61	1.63
CAG	4	0.11	GAG	14	0.37
CGU	13	0.93	GGU	48	0.91
CGC	0	0	GGC	10	0.19
CGA	47	1.17	GGA	108	2.06
AGG	0	0	GGG	44	0.84

*stop codon

Table S2 Relative synonymous codon usages (RSCUs) of PCGs of *Kiefferulus glauciventris*

Codon	Count	RSCU	Codon	Count	RSCU
UUU	395	1.8	AUU	320	1.78
UUC	43	0.2	AUC	39	0.22
UUA	391	4.09	AUA	179	1.63
UUG	37	0.39	AUG	40	0.37
UCU	129	3.21	ACU	89	1.97
UCC	11	0.27	ACC	11	0.24
UCA	79	1.97	ACA	76	1.61
UCG	7	0.17	ACG	8	0.18
UAU	117	1.67	AAU	188	1.76
UAC	23	0.33	AAC	26	0.24
UAA*	0	0	AAA	77	1.54
UAG*	0	0	AAG	23	0.46
UGU	31	1.94	AGU	44	1.1
UGC	1	0.06	AGC	7	0.17
UGA	87	1.81	AGA	43	1.07
UGG	9	0.19	AGG	1	0.02
CUU	70	0.73	GUU	92	1.94
CUC	12	0.13	GUC	13	0.27
CUA	54	0.57	GUA	72	1.52
CUG	9	0.09	GUG	13	0.27
CCU	70	2.14	GCU	97	2.31
CCC	25	0.76	GCC	23	0.55
CCA	34	1.04	GCA	43	1.02
CCG	2	0.06	GCG	5	0.12
CAU	55	1.38	GAU	50	1.49
CAC	25	0.63	GAC	17	0.51
CAA	65	1.78	GAA	64	1.66
CAG	8	0.22	GAG	13	0.34
CGU	13	0.93	GGU	45	0.86
CGC	2	0.14	GGC	16	0.3
CGA	37	2.64	GGA	99	1.89
CGG	4	0.29	GGG	50	0.95

*stop codon

Table S3 Relative synonymous codon usages (RSCUs) of PCGs of *Kiefferulus intertinctus*

Codon	Count	RSCU	Codon	Count	RSCU
UUU	390	1.81	AUU	349	1.87
UUC	41	0.19	AUC	24	0.13
UUA	419	4.42	AUA	187	1.73
UUG	31	0.33	AUG	29	0.27
UCU	132	3.31	ACU	71	1.58
UCC	11	0.28	ACC	11	0.24
UCA	77	1.93	ACA	93	2.07
UCG	3	0.08	ACG	5	0.11
UAU	129	1.77	AAU	180	1.69
UAC	17	0.23	AAC	33	0.31
UAA*	0	0	AAA	92	1.8
UAG*	0	0	AAG	10	0.2
UGU	32	1.88	AGU	50	1.25
UGC	2	0.12	AGC	9	0.23
UGA	88	1.83	AGA	37	0.93
UGG	8	0.17	AGG	0	0
CUU	73	0.77	GUU	100	2.19
CUC	2	0.02	GUC	8	0.17
CUA	41	0.43	GUA	69	1.51
CUG	3	0.03	GUG	6	0.13
CCU	73	2.25	GCU	86	2.04
CCC	19	0.58	GCC	19	0.45
CCA	32	0.98	GCA	59	1.4
CCG	6	0.18	GCG	5	0.12
CAU	62	1.61	GAU	58	1.71
CAC	15	0.39	GAC	10	0.29
CAA	68	1.81	GAA	71	1.87
CAG	7	0.19	GAG	5	0.13
CGU	16	1.14	GGU	59	1.11
CGC	2	0.14	GGC	7	0.13
CGA	34	2.43	GGA	100	1.89
AGG	4	0.29	GGG	46	0.87

*stop codon

Table S4 Relative synonymous codon usages (RSCUs) of PCGs of *Kiefferulus* sp.1XL

Codon	Count	RSCU	Codon	Count	RSCU
UUU	379	1.74	AUU	297	1.69
UUC	55	0.26	AUC	54	0.31
UUA	337	3.49	AUA	172	1.64
UUG	58	0.6	AUG	38	0.36
UCU	125	3.11	ACU	77	1.65
UCC	23	0.57	ACC	28	0.6
UCA	70	1.74	ACA	73	1.56
UCG	5	0.12	ACG	9	0.19
UAU	107	1.53	AAU	181	1.74
UAC	33	0.47	AAC	27	0.26
UAA*	0	0	AAA	81	1.59
UAG*	0	0	AAG	21	0.41
UGU	30	1.76	AGU	48	1.19
UGC	4	0.24	AGC	14	0.35
UGA	78	1.63	AGA	37	0.92
UGG	18	0.38	AGG	0	0
CUU	77	0.8	GUU	81	1.69
CUC	22	0.23	GUC	23	0.48
CUA	75	0.78	GUA	73	1.52
CUG	11	0.11	GUG	15	0.31
CCU	59	1.82	GCU	71	1.62
CCC	27	0.83	GCC	34	0.78
CCA	37	1.14	GCA	59	1.35
CCG	7	0.22	GCG	11	0.25
CAU	58	1.47	GAU	52	1.49
CAC	21	0.53	GAC	18	0.51
CAA	65	1.73	GAA	58	1.57
CAG	10	0.27	GAG	16	0.43
CGU	11	0.79	GGU	28	0.53
CGC	1	0.07	GGC	14	0.26
CGA	36	2.57	GGA	96	1.8
AGG	8	0.29	GGG	75	1.41

*stop codon

Table S5 Relative synonymous codon usages (RSCUs) of PCGs of *Kiefferulus trigonum*

Codon	Count	RSCU	Codon	Count	RSCU
UUU	429	1.91	AUU	361	1.87
UUC	20	0.09	AUC	25	0.13
UUA	435	4.69	AUA	213	1.86
UUG	20	0.22	AUG	16	0.14
UCU	145	3.6	ACU	86	1.9
UCC	10	0.25	ACC	10	0.22
UCA	70	1.74	ACA	82	1.81
UCG	1	0.02	ACG	3	0.07
UAU	124	1.8	AAU	188	1.77
UAC	14	0.2	AAC	25	0.23
UAA*	0	0	AAA	94	1.83
UAG*	0	0	AAG	9	0.17
UGU	31	1.88	AGU	41	1.02
UGC	2	0.12	AGC	4	0.1
UGA	87	1.79	AGA	51	1.27
UGG	10	0.21	AGG	0	0
CUU	64	0.69	GUU	85	1.93
CUC	5	0.05	GUC	5	0.11
CUA	29	0.31	GUA	83	1.89
CUG	3	0.03	GUG	3	0.07
CCU	78	2.4	GCU	90	2.29
CCC	4	0.12	GCC	8	0.2
CCA	47	1.45	GCA	54	1.38
CCG	1	0.03	GCG	5	0.13
CAU	63	1.73	GAU	64	1.86
CAC	10	0.27	GAC	5	0.14
CAA	72	1.95	GAA	70	1.87
CAG	2	0.05	GAG	5	0.13
CGU	10	0.71	GGU	50	0.96
CGC	2	0.14	GGC	5	0.1
CGA	39	2.79	GGA	115	2.21
AGG	5	0.32	GGG	38	0.73

*stop codon

Table S6 Relative synonymous codon usages (RSCUs) of PCGs of *Kiefferulus tainanus*

Codon	Count	RSCU	Codon	Count	RSCU
UUU	415	1.87	AUU	348	1.9
UUC	28	0.13	AUC	19	0.1
UUA	432	4.51	AUA	188	1.77
UUG	36	0.38	AUG	25	0.23
UCU	147	3.61	ACU	86	1.92
UCC	7	0.17	ACC	2	0.04
UCA	73	1.79	ACA	91	2.03
UCG	5	0.12	ACG	0	0
UAU	123	1.8	AAU	200	1.82
UAC	14	0.2	AAC	20	0.18
UAA*	0	0	AAA	87	1.76
UAG*	0	0	AAG	12	0.24
UGU	34	2	AGU	56	1.37
UGC	0	0	AGC	4	0.1
UGA	88	1.83	AGA	34	0.83
UGG	8	0.17	AGG	0	0
CUU	76	0.79	GUU	95	2.12
CUC	4	0.04	GUC	5	0.11
CUA	24	0.25	GUA	74	1.65
CUG	3	0.03	GUG	5	0.11
CCU	69	2.12	GCU	77	1.83
CCC	16	0.49	GCC	14	0.33
CCA	44	1.35	GCA	71	1.69
CCG	1	0.03	GCG	6	0.14
CAU	59	1.55	GAU	58	1.71
CAC	17	0.45	GAC	10	0.29
CAA	69	1.82	GAA	67	1.79
CAG	7	0.18	GAG	8	0.21
CGU	7	0.5	GGU	45	0.86
CGC	1	0.17	GGC	3	0.06
CGA	44	3.14	GGA	117	2.23
AGG	4	0.29	GGG	45	00.86

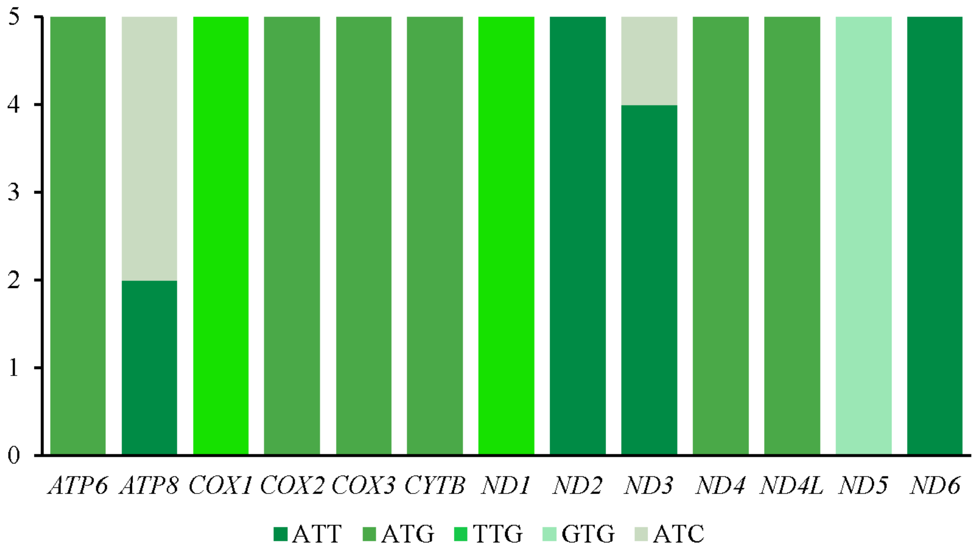

Figure S1. Start codons of protein-coding genes among *Kiefferulus* mitogenomes

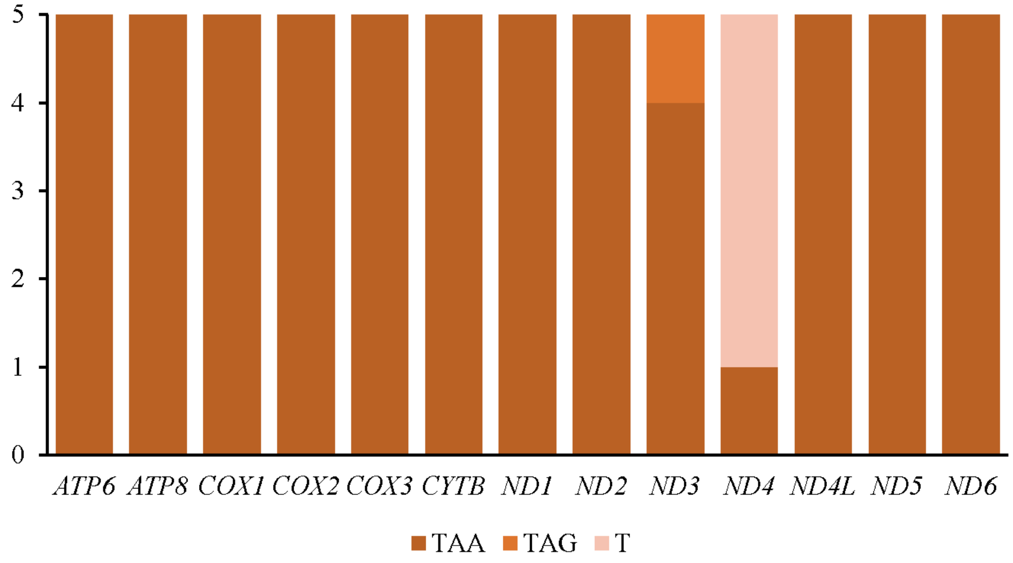

Figure S2. Stop codon of protein-coding genes among *Kiefferulus* mitogenomes

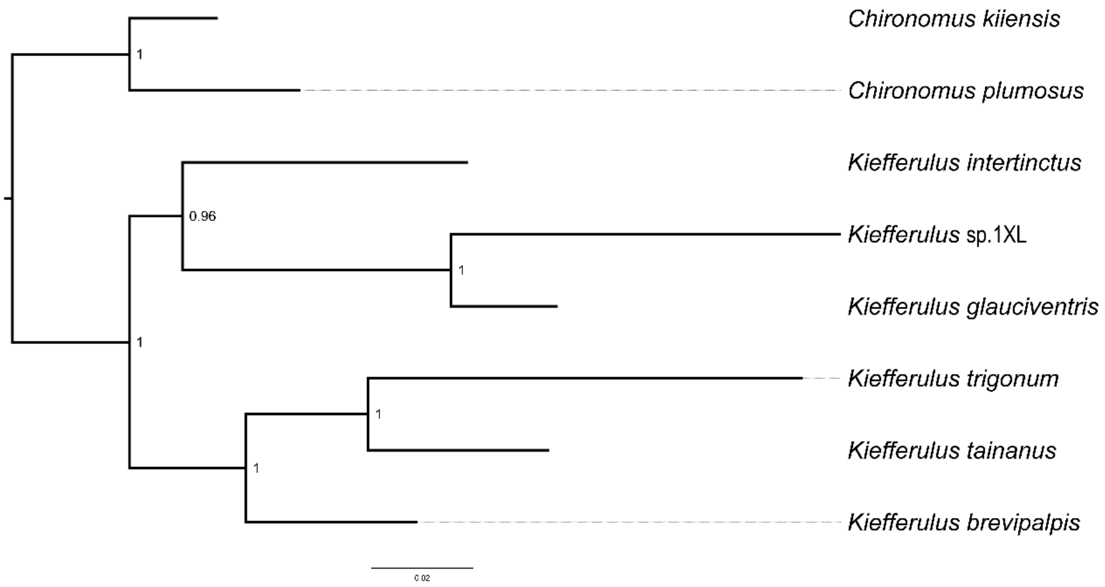

Figure S3. Bayesian inference phylogenetic tree of *Kiefferulu* based on the analysis cd_faa with a GTR + CAT model in phylobayes. Support values on nodes indicate Bayesian posterior probabilities.

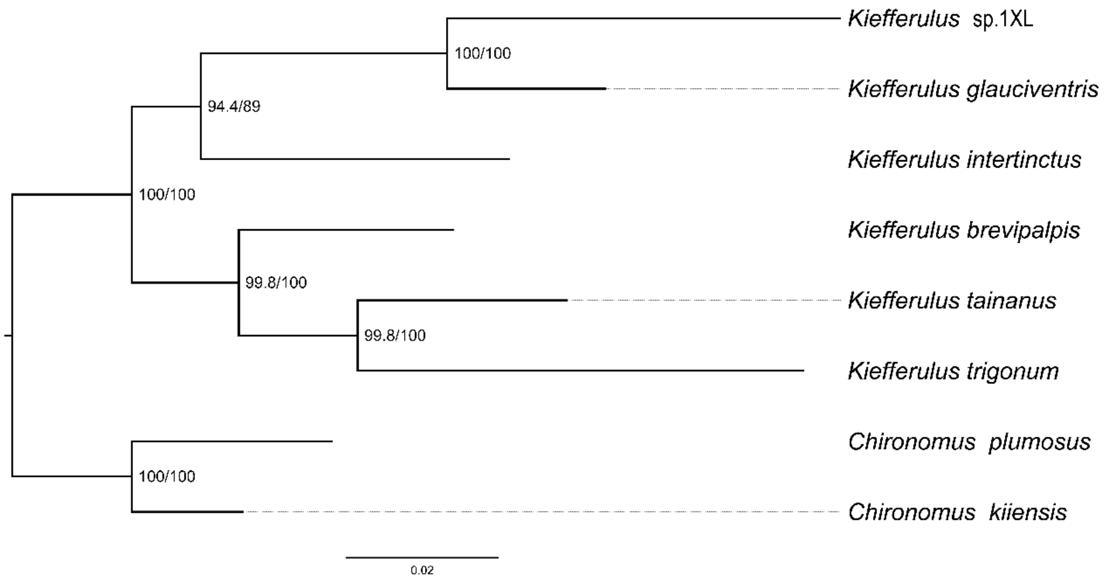

Figure S4. Maximum likelihood phylogenetic tree of *Kiefferulu* based on the analysis cds_faa with PMSF model in IQTREE. Support values on nodes indicate SH-aLRT/UFBoot2, respectively.

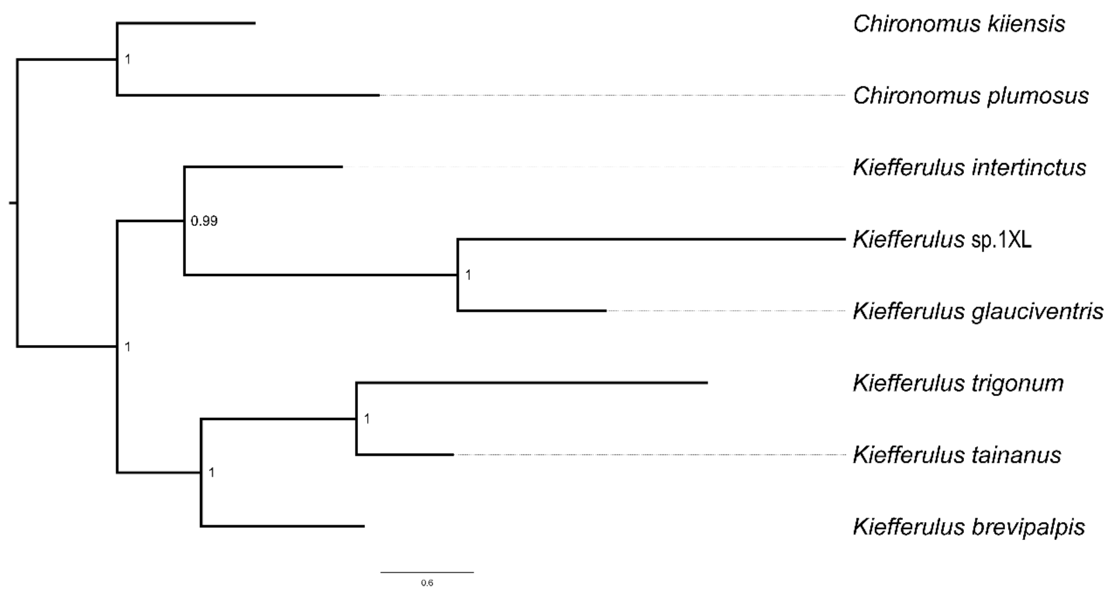

Figure S5. Bayesian inference phylogenetic tree of *Kiefferulu* based on the analysis cd_fna with a GTR + CAT model in phylobayes. Support values on nodes indicate Bayesian posterior probabilities.

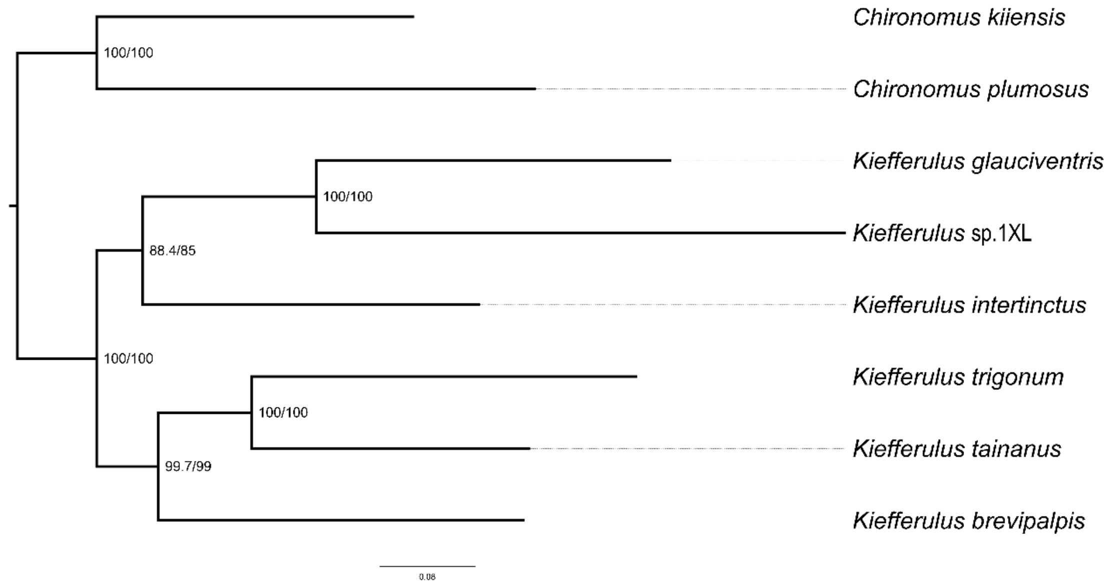

Figure S6. Maximum likelihood phylogenetic tree of *Kiefferulu* based on the analysis cds_fna with a Partitioned model in IQTREE. Support values on nodes indicate SH-aLRT/UFBoot2, respectively.

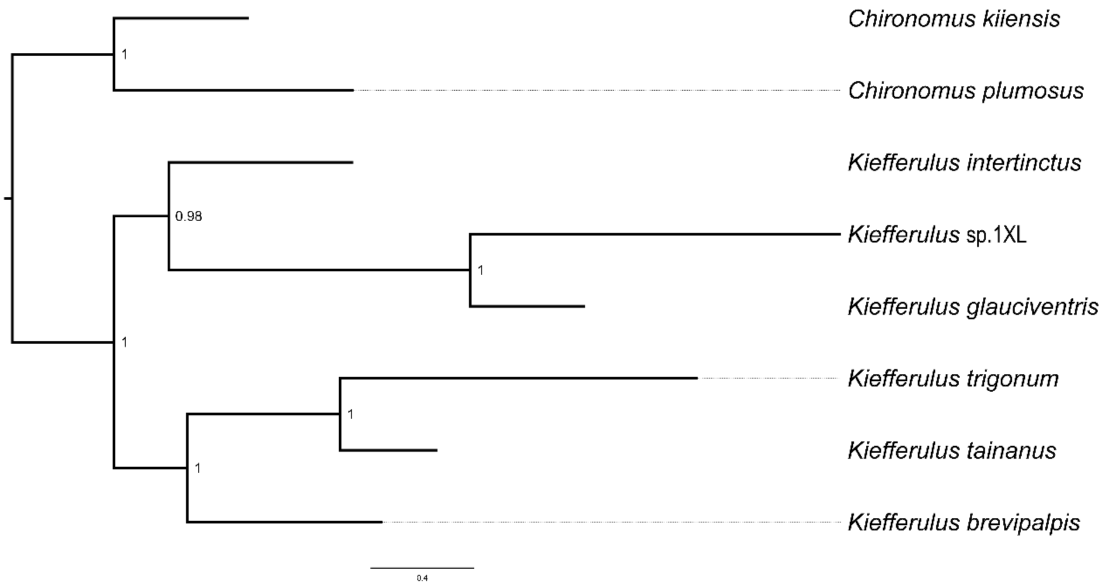

Figure S7. Bayesian inference phylogenetic tree of *Kiefferulu* based on the analysis cd_rrna with a GTR + CAT model in phylobayes. Support values on nodes indicate Bayesian posterior probabilities.

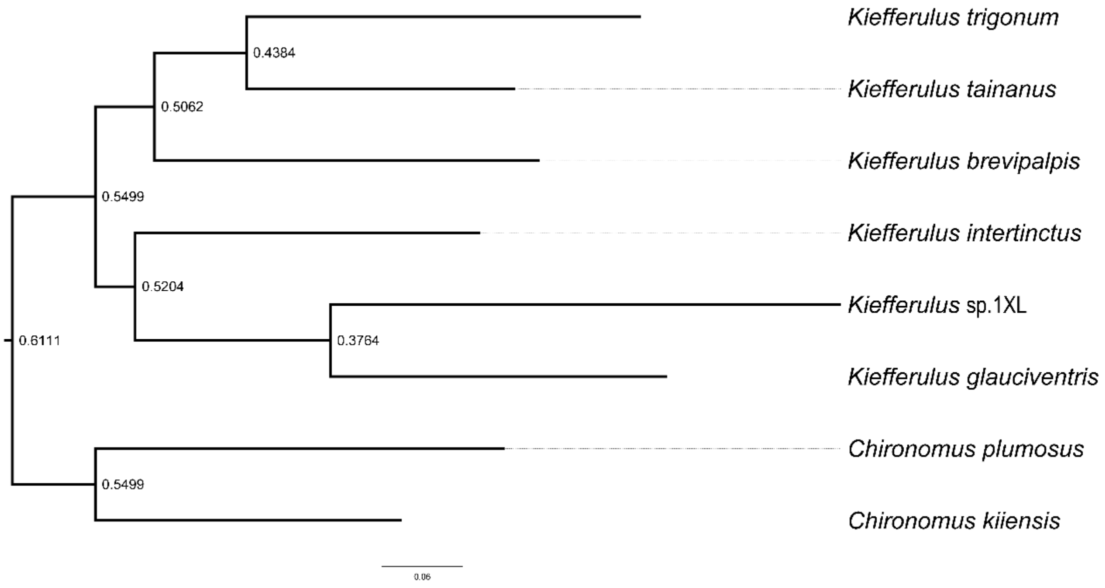

Figure S8. Maximum likelihood phylogenetic tree of *Kiefferulu* based on the analysis cds_rrna with a Partitioned model in IQTREE. Support values on nodes indicate SH-aLRT/UFBoot2, respectively.

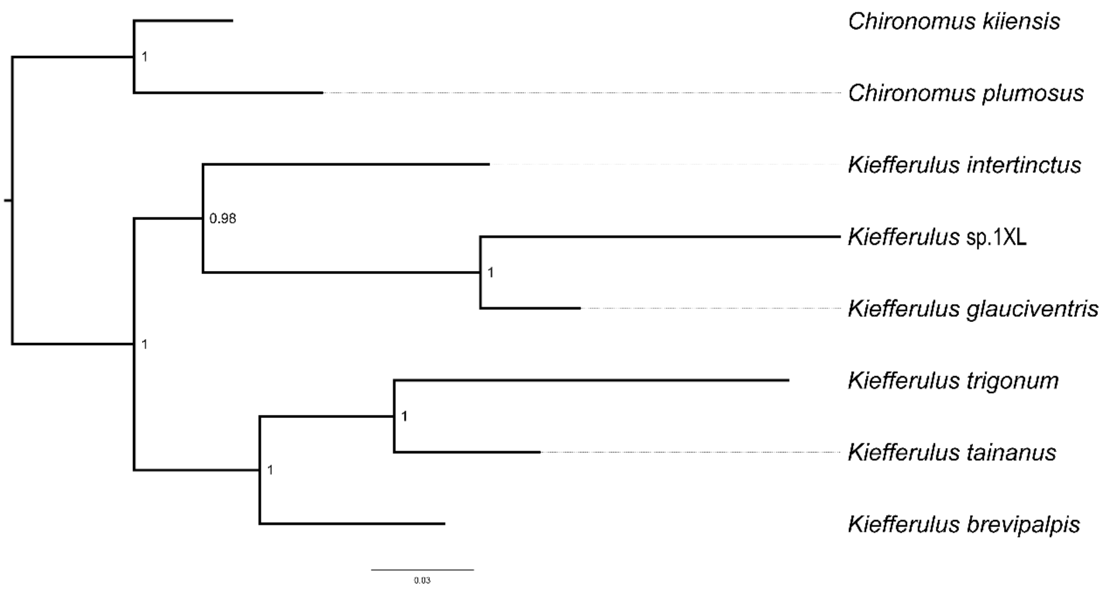

Figure S9. Bayesian inference phylogenetic tree of *Kiefferulu* based on the analysis cd12_fna with a GTR + CAT model in phylobayes. Support values on nodes indicate Bayesian posterior probabilities.

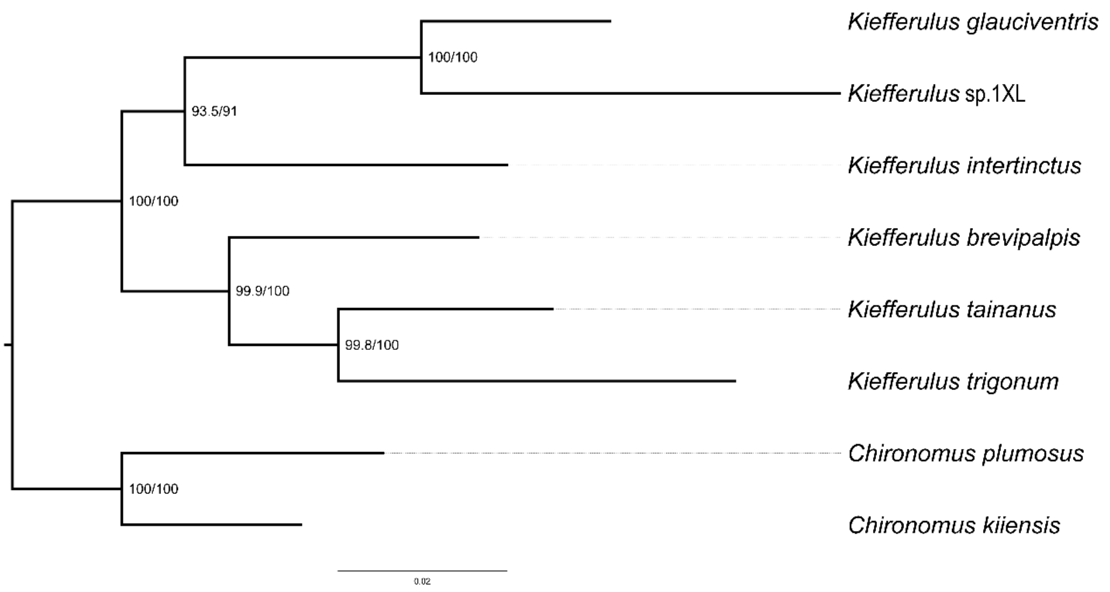

Figure S10. Maximum likelihood phylogenetic tree of *Kiefferulu* based on the analysis cds12_fna with a Partitioned model in IQTREE. Support values on nodes indicate SH-aLRT/UFBoot2, respectively.

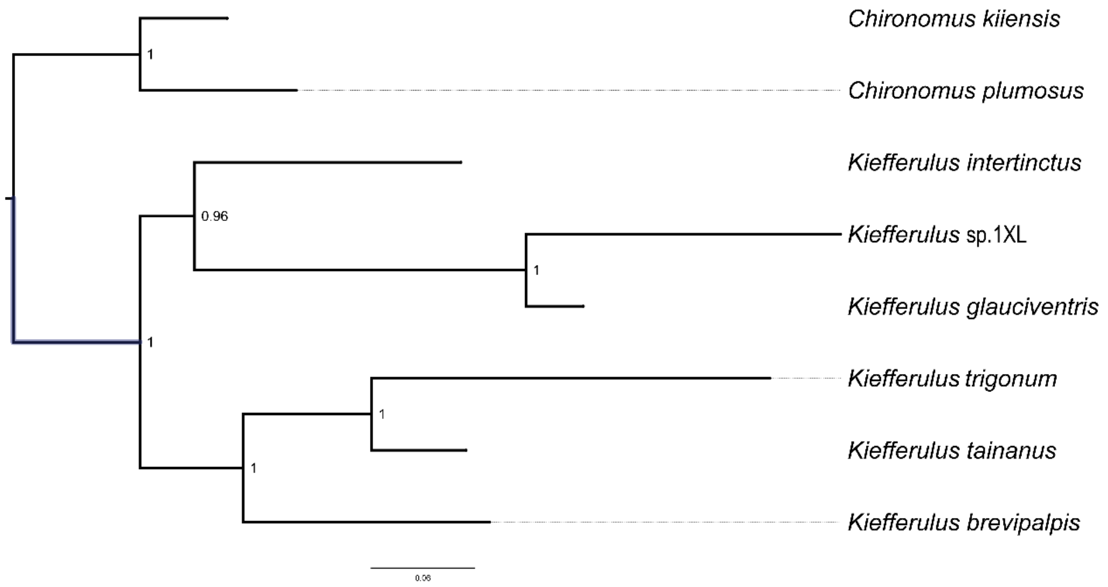

Figure S11. Bayesian inference phylogenetic tree of *Kiefferulu* based on the analysis cd12_rrna with a GTR + CAT model in phylobayes. Support values on nodes indicate Bayesian posterior probabilities.

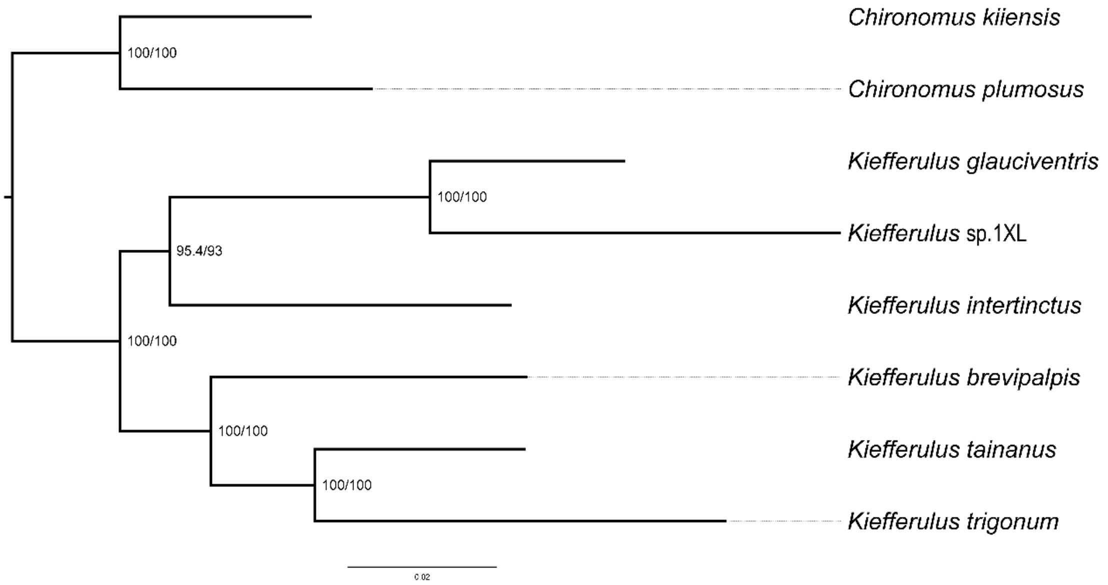

Figure S12. Maximum likelihood phylogenetic tree of *Kiefferulu* based on the analysis cds12_rrna with a Partitioned model in IQTREE. Support values on nodes indicate SH-aLRT/UFBoot2, respectively.
